# A Systematic Review on Power Estimation and Sample Size Calculation in Randomized Controlled Trials of Surgical Treatments for Benign Prostatic Hyperplasia

**DOI:** 10.7759/cureus.96843

**Published:** 2025-11-14

**Authors:** Daniel Akintelure, Regina U Agada, Simon Akintelure, Don S Wijayasuriya, Philip Abolanle

**Affiliations:** 1 Urology, Royal Gwent Hospital, Newport, GBR; 2 Medicine, University of Warwick, Coventry, GBR; 3 Medicine, All Saints University School of Medicine Dominica, Roseau, DMA

**Keywords:** benign prostatic hyperplasia (bph), power test, randomized clinical trial, statistic, urology surgery

## Abstract

Randomized controlled trials (RCTs) are considered the gold standard for evaluating surgical interventions for benign prostatic hyperplasia (BPH). However, the validity and reliability of their findings depend critically on adequate sample size determination and power estimations. Inadequate or absent reporting of these parameters can undermine the interpretability of trial outcomes. This systematic review aimed to evaluate the prevalence, methodological rigor, and temporal trends of sample size calculation (SSC) and power reporting in RCTs of surgical interventions for BPH. We conducted a comprehensive systematic search of PubMed and the Cochrane Central Register of Controlled Trials (CENTRAL). Eligible studies were RCTs that investigated surgical treatments for BPH and reported relevant clinical outcomes. Data extraction captured whether SSC and power estimation were reported, along with details of alpha levels, power values, year of publication, and intervention type. Subgroup analyses were performed according to intervention category, and associations were tested using Pearson’s chi-square test with ORs and 95% CIs.

Fifty-six RCTs were included. Of these, 30 trials (53.6%) reported performing SSC and power estimation, whereas 26 (46.4%) did not. Among those who reported SSC, most adopted a type I error threshold of 0.05 and a power value of at least 80%. Reporting practices demonstrated temporal improvement: SSC was documented in 33.3% of trials published between 1991 and 2000, 22.2% between 2001 and 2010, 64.0% between 2011 and 2020, and 57.9% between 2021 and 2025. Subgroup analysis revealed higher SSC reporting in novel minimally invasive surgical therapy (nMIST) trials than in non-MIST trials (62.5% vs. 31.3%, respectively). The association between intervention type and SSC reporting was statistically significant (χ²(1) = 4.49, p = 0.034), with the odds of SSC reporting being greater in nMIST-involved trials (OR 3.67, 95% CI 1.07-12.62). This review demonstrates gradual but meaningful improvements in SSC and power reporting in surgical RCTs for BPH over the past three decades, particularly in trials investigating minimally invasive techniques. Nonetheless, nearly half of the published trials continue to omit these critical methodological details, raising concerns about the robustness of their conclusions. Enhanced adherence to reporting standards, stricter editorial requirements, and improved researcher training are needed to ensure that future RCTs provide transparent and reproducible evidence to guide clinical practice.

## Introduction and background

Benign prostatic hyperplasia (BPH) is a non-malignant growth or enlargement of prostate tissue and a leading cause of lower urinary tract symptoms (LUTS) in aging men [1]. Several definitions of BPH exist in the literature, including bladder outlet obstruction, LUTS, and benign prostatic enlargement (BPE) [2]. Clinically, BPH can be described as one or more prostate adenomas causing varying degrees of bladder outlet obstruction [1]. Another perspective describes BPH as histological changes within the prostate gland, which involve microscopic proliferation of epithelial and stromal cells [2]. BPE refers to an increase in prostate size, usually secondary to BPH, while bladder outlet obstruction refers to impaired urinary flow. Patients with BPE who present with obstruction are classified as having benign prostatic obstruction (BPO) [2]. The prevalence of BPH increases with age, with histological studies reporting BPH in 50-60% of men in their 60s and rising to 80-90% of those older than 70 years [3]. Given the high prevalence and clinical burden of BPH, several therapeutic approaches have been developed.

Both surgical and non-surgical treatments are available for BPH, with selection based on symptom severity, patient preference, and overall health [4]. Medical therapy is frequently used as first-line management, whereas surgical intervention remains the standard of care for patients with moderate-to-severe symptoms or those unresponsive to medication [5]. Surgical treatments such as transurethral resection of the prostate (TURP) and newer minimally invasive procedures provide effective relief for moderate to severe symptoms, while medical therapy and lifestyle modification are generally suited for mild cases [6]. Traditional options such as TURP and open prostatectomy have been complemented by newer minimally invasive techniques, including holmium laser enucleation (HoLEP), thulium laser enucleation (ThuLEP), photovaporization (PVP), and aquablation, which aim to reduce postoperative morbidity [7].

With the growing number of available surgical options, high-quality comparative evidence is essential for guiding clinical decision-making. Randomized controlled trials (RCTs) are the most reliable method for comparing surgical approaches and medical managements [8, 9]. They are considered essential for evaluating the efficacy and safety of interventions in evidence-based medicine, providing solid evidence of the effects of different treatments [9]. These trials help determine which treatments offer the best outcomes, including symptom relief, reduced complications, and improved quality of life while minimizing risks, such as sexual dysfunction. However, these trials may yield biased or unreliable results if they lack methodological rigor, particularly regarding power estimation and sample size calculation (SSC) [10].

Despite their importance, many RCTs evaluating BPH treatments suffer from methodological shortcomings, including concerns over the reliability and generalizability of their findings. Common issues include inadequate blinding, short follow-up periods that fail to capture long-term outcomes, heterogeneous selection criteria, and inconsistent definitions or measurements of clinical endpoints such as urinary flow rates and symptom scores. Additionally, small sample sizes, selective outcome reporting, and insufficient transparency in statistical planning are common limitations. These weaknesses may lead to overestimation of treatment benefits or underestimation of adverse effects, ultimately hindering clinicians’ ability to draw reliable conclusions about comparative efficacy and safety.

Power estimation and SSC are essential for ensuring that trials are adequately powered to detect clinically meaningful differences while avoiding unnecessary participant exposure and excessive resource use [11]. Appropriate SSC requires pre-specifying the type I error rate, type II error rate (power), minimal clinically important difference (MCID), expected variability, and considering feasibility, available resources, and anticipated dropout rates [12-17]. Insufficient sample sizes increase the risk of type II errors and falsely negative results, whereas excessively large samples increase costs, prolong study duration, and expose more participants than necessary. Transparent SSC reporting is therefore critical for evaluating RCT validity and reproducibility.

Because it is rarely feasible to study an entire population, clinical research is typically conducted on samples drawn from that population [15]. Therefore, the calculation of an appropriate sample size is fundamental to study design, as it directly determines the precision, validity, and interpretability of trial findings. Studies with insufficient sample sizes are at risk of type II errors, whereby a true effect is missed and the trial yields a falsely negative result. Although smaller sample sizes may appear attractive because of lower costs and logistical simplicity, they ultimately render studies inconclusive and represent a waste of resources. Conversely, excessively large sample sizes are discouraged, as they can lead to unnecessary expenditure, prolonged study duration, and exposure of more participants than required to potential risks [16]. Thus, careful SSC is essential and should be informed by the study objectives, data types, and distributional assumptions [15].

Determining the appropriate sample size is a fundamental step in the design of clinical research, as it is often the most critical factor influencing both the duration and financial cost of a study [15]. A valid and transparent SSC requires several essential components. First, the probability of committing a type I error must be specified, which represents the risk of incorrectly concluding that a treatment effect exists when it does not. Second, the probability of a type II error should be defined, which reflects the risk of failing to detect a true effect and concluding that no meaningful difference exists between groups. In addition, researchers must establish the MCID, which is the smallest difference in outcomes considered relevant for clinical decision-making and, therefore, worth detecting. Finally, an estimate of the variability in the population, often expressed as the standard deviation, is required to inform the precision of the study estimates [17].

Moreover, SSCs must consider all available data, funding, support facilities, and ethical considerations related to subjecting patients to research. The final number should be increased to account for a safety margin and anticipated dropout rate [15].

The growing need for evidence-based medical practice has generated an increasing amount of medical literature supported by statistical methods, and to assess a trial accurately, readers of published reports require complete, clear, and transparent information on study methodology and findings [13,18]. Unfortunately, many published RCTs on surgical BPH management fail to provide adequate reporting of SSC or power estimation, raising concerns about whether these trials were appropriately designed to detect meaningful clinical differences.

Although the reporting of SSCs has increased over the past two decades, only about one-third (34%) of SSC descriptions have been adequately reported, even in high-impact general medical journals [11]. Given the implications of underpowered or poorly planned studies for clinical decision-making, there is a need to systematically assess the prevalence, accuracy, and methodological rigor of SSC and power estimation in RCTs evaluating the surgical management of BPH. Therefore, this systematic review aimed to evaluate the extent to which RCTs on surgical interventions for BPH report SSC and power estimations, assess the methodological rigor of these reports, and explore how reporting practices have evolved over time and across intervention types.

## Review

Methods

*Approach* 

This systematic review was conducted in accordance with the Preferred Reporting Items for Systematic Reviews and Meta-Analyses (PRISMA) guidelines [19]. The primary objective of this review was to evaluate RCTs investigating surgical interventions for BPH, with a specific focus on whether power estimations and SSCs were reported, and the extent to which these were methodologically rigorous.

Eligibility Criteria

The eligibility criteria were defined a priori to ensure consistency and transparency of the study. Studies were included if they were RCTs involving adult male participants diagnosed with BPH and treated with surgical interventions such as TURP, HoLEP, ThuLEP, PVP, or aquablation. Trials were required to report clinical outcomes relevant to BPH management. Studies were excluded if they were non-randomized, observational, retrospective, or case studies, or if they focused exclusively on medical or minimally invasive non-surgical therapies such as UroLift or Rezūm. Review articles, study protocols, editorials, and conference abstracts without available full-text data were also excluded.

Search Strategy

A comprehensive literature search was conducted in PubMed, Embase, and CENTRAL. The search strategy employed a combination of keywords and Medical Subject Headings (MeSH) related to “benign prostatic hyperplasia,” “surgical treatment,” “randomized controlled trial,” “sample size calculation,” and “power estimation.” To minimize the risk of missing eligible studies, the reference lists of the included articles were manually screened for additional records.

Study Selection 

All records retrieved from the databases were imported into Rayyan (Rayyan Systems Inc., Doha, Qatar) to facilitate screening [20]. Duplicate records were removed, and the studies were screened in two stages. First, the titles and abstracts were reviewed to exclude irrelevant records, non-RCTs, and studies not focused on surgical interventions. Second, the full texts of potentially eligible studies were assessed against the predefined inclusion and exclusion criteria. Two reviewers independently screened the studies at both stages, and disagreements were resolved through discussion and consensus. Two review authors (R.U.A. and S.A.) independently extracted and recorded the data using a predefined checklist. In the case of disagreements, a third review author (D.A.) was involved, and a consensus was reached.

Data Extraction

Data extraction was performed using a standardized template in Microsoft Excel. The extracted variables included study characteristics (such as year of publication, country, and type of surgical intervention), whether a SSC was performed, the reported power, the assumed type I error, and the MCID, where available. Additional trial features, such as follow-up duration and outcome measures, were recorded to provide context for methodological assessment. A fourth author (D.S.W.) reviewed the completed data extraction form and checked it against the included studies.

For the purpose of this review, novel minimally invasive surgical therapy (nMIST) was defined as procedures designed to reduce morbidity and recovery time compared with conventional TURP or open prostatectomy. Specifically, nMIST included HoLEP, ThuLEP, PVP, aquablation, and similar laser-based or minimally invasive approaches. Conventional surgery encompassed TURP (monopolar or bipolar) and open prostatectomy. These classifications were determined a priori based on definitions used in previous urological systematic reviews.

Risk of Bias Assessment

The methodological quality of the included trials was appraised using the Cochrane Risk of Bias 2 tool, which evaluates bias across domains such as the randomization process, deviations from intended interventions, missing outcome data, measurement of outcomes, and selective reporting [21]. This assessment was conducted by a review author (D.A.).

Data Synthesis and Analysis

Descriptive statistics were used to summarize the reporting of SSCs, power values, and related statistical assumptions. Frequencies and percentages were calculated for categorical variables, while continuous variables were summarized using means or medians, as appropriate, to provide a clear overview of reporting patterns. The rationale for using descriptive methods was to capture the prevalence and distribution of reporting practices rather than estimating effect sizes across heterogeneous interventions. Pearson’s chi-square tests were performed to examine associations between intervention type and the likelihood of reporting SSCs. This non-parametric test was selected because it is well suited for categorical data and allows the assessment of whether observed differences in reporting across intervention categories were statistically significant beyond random variation. ORs with corresponding 95% CIs were calculated to quantify the strength of the association. All statistical analyses were performed using Microsoft Excel, and the results are presented in tabular and graphical forms to highlight patterns and temporal trends in reporting practices among RCTs of BPH surgery.

Results

**Figure 1:**
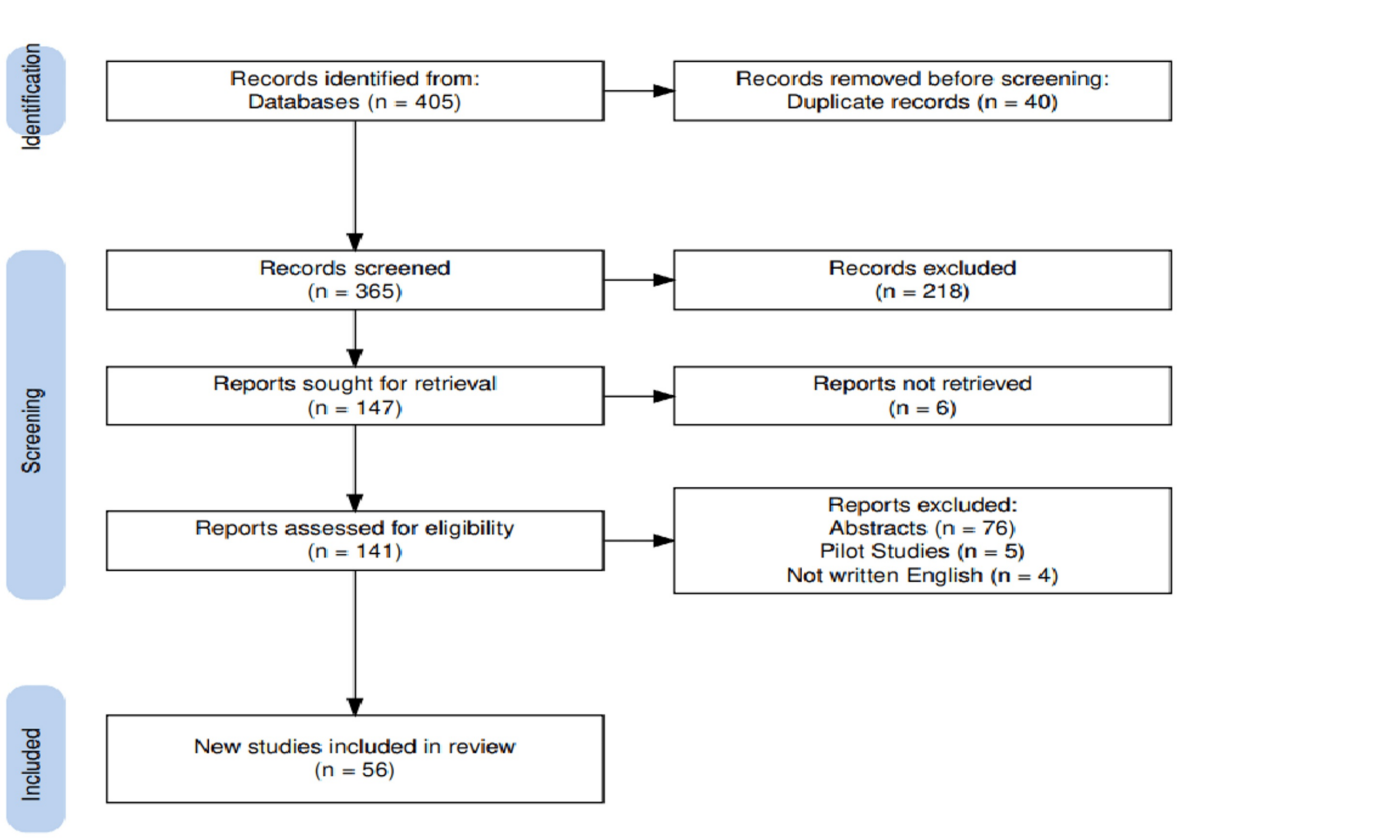
Flow diagram illustrating the identification, screening, eligibility assessment, and inclusion of studies in accordance with PRISMA guidelines for this systematic review. PRISMA: Preferred Reporting Items for Systematic Reviews and Meta-Analyses.

The database search identified 405 records screened, as shown in Figure 1. After the removal of 40 duplicates, 365 records remained and were screened by title and abstract. Title and abstract screening excluded 218 studies (143 review articles, 62 non-surgical intervention studies, and 13 non-RCTs). Of the 147 articles remaining, six were excluded due to no response from authors regarding ambiguous reporting, leaving 141 articles for full-text assessment. Of these, 85 were excluded (76 were published only as abstracts and lacked sufficient information, five were pilot studies, and four were not in English). Finally, 56 studies were included. The full details of the study characteristics are shown in Table 1.

**Table 1:** Study characteristics. TPLA: Transperineal Laser Ablation; TURP: Transurethral Resection of the Prostate; IPSS: International Prostate Symptom Score; PUL: Prostatic Urethral Lift; PVP: Photoselective Vaporization of the Prostate; PSEP: Photoselective Sharp Enucleation of the Prostate; PAE: Prostatic Artery Embolization; HoLXV: Holmium Laser Xpeeda Vaporization; GL-XPS: GreenLight XPS Laser Vaporization; TUVP: Transurethral Electrovaporization of the Prostate; ThuVARP: Thulium Laser Transurethral Vapo-Resection of the Prostate; Qmax: Maximum Urinary Flow Rate; DiLEP: Diode Laser Enucleation of the Prostate; PKRP: Plasmakinetic Resection of the Prostate; HoLEP: Holmium Laser Enucleation of the Prostate; PKVP: Plasmakinetic Vaporesection of the Prostate; H-FIRE: High-Frequency Irreversible Electroporation; TUIBN: Transurethral Incision of the Bladder Neck; SHIM: Sexual Health Inventory for Men; SP: Suprapubic Prostatectomy; RP: Retropubic Prostatectomy; ThuLEP: Thulium Laser Enucleation of the Prostate; ThuVEP: Thulium Vapoenucleation of the Prostate; BPKEP: Bipolar Plasmakinetic Enucleation of the Prostate; BPV: Bipolar Plasma Vaporization; TUDP: Transurethral Detachment Prostatectomy; BPVP: Bipolar Plasma Vaporization of the Prostate; C-BPVP: Continuous Bipolar Plasma Vaporization of the Prostate; S-BPVP: Standard Bipolar Plasma Vaporization of the Prostate; HRQL: Health-Related Quality of Life; PVR: Post-Void Residual; AE: Antegrade Ejaculation; QoL: Quality of Life; IIEF-15: International Index of Erectile Function–15; TFL: Thulium Fiber Laser; TmLRP: Thulium Laser Resection of the Prostate; AUA-7: American Urological Association Symptom Score (7-item); EJ-MSHQ: Ejaculatory Domain of the Male Sexual Health Questionnaire; CISC: Clean Intermittent Self-Catheterization; RB-TVP: Rectal Balloon Transvesical Prostatectomy; KTP-PVP: Potassium Titanyl Phosphate Photoselective Vaporization of the Prostate; OP: Open Prostatectomy.

Author (Year)	Country/Region	Sample Size	Intervention	Control	Outcome Measures	Alpha Value	Power Value	Effect Size / Key Findings
Zhang W et al. (2021) [22]	China	119	TPLA	TURP	IPSS	0.05	80%	No significant difference (p > 0.05)
Sønksen J et al. (2015) [23]	Europe	80	TURP	PUL	Responder Rate (BPH6)	0.05	80%	RR = 4.06; RD = 26.3%; OR = 5.70
Gilling P et al. (2018) [24]	USA, UK, Australia, New Zealand	181	Aquablation	TURP	Anejaculation	0.05	80%	RD = –16.0 pp; RR = 0.62; OR ≈ 0.49; p = 0.0149
Liu Z et al. (2022) [25]	China	154	PSEP	PVP	IPSS	NR	-	No significant difference
Abt D et al. (2021) [26]	Switzerland	103	PAE	TURP	IPSS	0.05	80%	Mean difference = 2.88
Elmansy H et al. (2023) [27]	Egypt	92	HoLXV	GL-XPS	IPSS	0.05	90%	No significant difference (p > 0.05)
Fowler C et al. (2005) [28]	England	235	TUVP	TURP	IPSS	0.05	80%	No significant difference (p > 0.05)
Worthington J et al. (2020) [29]	England	410	ThuVARP	TURP	IPSS, Qmax	0.05	85%	Equivalent IPSS improvement; TURP superior for Qmax (mean difference = 3.12 mL/s; 95% CI 0.45–5.79)
Zhang J et al. (2019) [30]	NR	157	DiLEP	PKRP	IPSS	0.05	-	No significant difference (p > 0.05)
Müllhaupt G et al. (2021) [31]	Switzerland	120	Aquablation	HoLEP	IPSS	0.05	90%	No significant difference (p > 0.05)
Abt D et al. (2014) [32]	Switzerland	103	PAE	TURP	IPSS	0.05	80%	Mean difference = 2.88
Carneiro A et al. (2016) [33]	Brazil	78	Suprapubic (SP)	Retropubic (RP)	IPSS	0.05	80%	No significant difference
Gao Z et al. (2024) [34]	China	87	LP-HoLEP	HP-HoLEP	IPSS	0.05	80%	No significant difference
Fung BT et al. (2005) [35]	China	60	PKVP	TURP	IPSS	NR	-	No significant difference
He BM et al. (2025) [36]	China	118	H-FIRE	TURP	IPSS	0.025	90%	No significant difference (p > 0.025)
Montorsi F et al. (2008) [37]	NR	100	HoLEP	TURP	IPSS	NR	NR	No significant difference
Michielsen DP et al. (2007) [38]	NR	236	B-TURP	M-TURP	Serum sodium & chloride	NR	NR	Significantly greater changes in M-TURP
Li X et al. (2013) [39]	NR	124	STURP + TUIBN	TURP	IPSS	NR	NR	No significant difference
Telli O et al. (2015) [40]	Turkey	101	PVP	TURP	IPSS, SHIM, Qmax	NR	NR	Comparable outcomes; PVP had shorter hospital stay (p < 0.001)
Xu C et al. (2019) [41]	China	200	Two-Lobe HoLEP	Three-Lobe HoLEP	IPSS	NR	NR	No significant difference
Worthington J et al. (2017) [42]	UK	420	ThuVARP	TURP	IPSS	0.05	85%	No significant difference
Fuschi A et al. (2021) [43]	Italy	110	HoLEP	LSP & RASP	Functional & perioperative outcomes	NR	NR	No significant differences except longer catheterization in LSP (p = 0.002)
Kazemi R et al. (2023) [44]	Iran	54	Prostatectomy (+ Surgicel)	Prostatectomy (no Surgicel)	IPSS	NR	NR	No significant difference
Zou Z et al. (2018) [45]	China	114	DiLEP	BPKEP	IPSS, Qmax	0.025	80%	No significant difference
Zhang J et al. (2020) [46]	China	116	HoLEP	ThuLEP	IPSS, Qmax	0.05	80%	No significant difference
Paighan N et al. (2024) [47]	India	68	ThuVEP	TURP	IPSS, Qmax	NR	NR	ThuVEP improved Qmax; IPSS comparable
Skinner TA et al. (2017) [48]	Canada	55	Laser Vaporization	BPV	IPSS	0.05	90%	No significant difference
Netsch C et al. (2017) [49]	Germany	107	ThuVEP	HoLEP	IPSS	0.05	90%	No significant difference
Pan C et al. (2020) [50]	China	113	TUDP	TURP	IPSS	0.05	90%	No significant difference
Nguyen DD et al. (2021) [51]	Multinational	-	Aquablation	TURP	IPSS	NR	NR	No significant difference
Masood A et al. (2025) [52]	India	110	B-TURP	M-TURP	Serum sodium	0.05	80%	Bipolar: -1.8 vs Monopolar -5.1 mEq/L (p < 0.05)
Okada T et al. (2022) [53]	NR	71	PVP	DVP & ThuARP	QOL index, Qmax	NR	NR	Vaporization rate highest in PVP (p < 0.05)
Kumar N et al. (2018) [54]	India	186	PVP	B-TURP / M-TURP	IPSS	NR	NR	Sustained improvement; no significant differences
Liu CK et al. (2006) [55]	Taiwan	76	TUVRP	TURP	Catheterization duration	NR	NR	Shorter duration in TUVRP (p < 0.0001)
Nuhoğlu B et al. (2006) [56]	Turkey	57	PKRP	TURP	Catheterization time	NR	NR	PKRP shorter (42 h vs 75.7 h; p < 0.0001)
Hamouda A et al. (2014) [57]	Egypt	60	HoLEP	TURP	Hemoglobin loss	NR	NR	Comparable values (specific numbers not provided)
Riehmann M et al. (1995) [58]	USA	120	TUIP	TURP	IPSS, Qmax	NR	NR	Both improved; higher retrograde ejaculation after TURP (68% vs 35%, p = 0.020)
Gao Z et al. (2025) [59]	China	180	Single-n / Double-n	HoLEP	AE rate	NR	NR	AE highest in Double-n (77.8%) (p < 0.05)
Elmansy H et al. (2024) [60]	Egypt	100	Top-down HoLEP	HoLEP	IPSS, Qmax, QoL, PVR, IIEF-15	0.05	90%	No significant difference
Elmansy H et al. (2022) [61]	Canada	82	MOSES™	TFL	Operative efficiency, IPSS, QoL, Qmax, PVR	0.05	90%	MOSES™ shorter procedure; no postoperative differences
Cui D et al. (2014) [62]	China	96	TmLRP	TURP	IPSS, QoL, Qmax, PVR	0.05	80%	Comparable outcomes at all time points
Gilling PJ et al. (2022) [63]	New Zealand	181	Aquablation	TURP	IPSS	0.025	80%	Significant IPSS improvement in both groups; non-inferior; advantage in larger prostates
Keoghane SR et al. (1996) [64]	UK	148	Contact laser vaporization	TURP	AUA-7 score	0.05	90%	No significant difference
Bertolo R et al. (2023) [65]	Italy	55	TPLA	TURP	EJ-MSHQ	0.05	90%	No significant change in TPLA; TURP reduced ejaculatory function
Plante M et al. (2019) [66]	Multinational	-	Aquablation	TURP	IPSS	NR	NR	Greater IPSS reduction in large prostates; fewer complications and anejaculation
Geavlete B et al. (2014) [67]	Romania	180	C-BPVP	BPVP & TURP	IPSS, Qmax	0.05	85%	Comparable outcomes; shorter operation and fewer complications in C-BPVP
Muslumanoglu AY et al. (2012) [68]	Turkey	101	PKRP	TURP	IPSS	NR	NR	Long-term outcomes comparable (p = 0.34)
Dall’Oglio MF et al. (2006) [69]	Brazil	62	Retropubic	Transvesical	Blood loss, Hb drop	NR	NR	Significantly lower blood loss and Hb drop (p < 0.001)
Chen YB et al. (2013) [70]	China	280	PKRP	HoLEP	IPSS, QoL, Qmax	0.05	90%	Similar efficacy; HoLEP had less bleeding & shorter stay
Nielsen HO (1988) [71]	Denmark	49	TUT	TURP	Qmax, symptom scores	0.05	90%	Comparable improvement; TUT had shorter operative time
Ghalayini IF et al. (2005) [72]	UK	41	CISC before TURP	TURP alone	IPSS, QoL	NR	NR	Both improved; CISC improved bladder function (p < 0.001)
Kasivisvanathan V et al. (2018) [73]	USA	90	Aquablation	TURP	IPSS	-	90%	No significant difference (p = 0.7117)
Kumar S et al. (2025) [74]	India	80	B-TURP	M-TURP	Qmax, IPSS	NR	NR	B-TURP reduced blood loss (p < 0.001) with similar outcomes
Mohyelden K et al. (2015) [75]	Egypt	100	RB-TVP	TVP	Hb loss, irrigation use, stay	NR	NR	All perioperative outcomes favored RB-TVP (p ≤ 0.001)
Purkait B et al. (2017) [76]	Turkey	150	KTP-PVP	TURP	IPSS, Qmax	0.05	80%	Sustained improvement in both; higher retrograde ejaculation in TURP
Simforoosh N et al. (2010) [77]	Iran	100	OP	TURP	Qmax	0.05	90%	Greater Qmax improvement in OP (p = 0.02)

**Figure 2:**
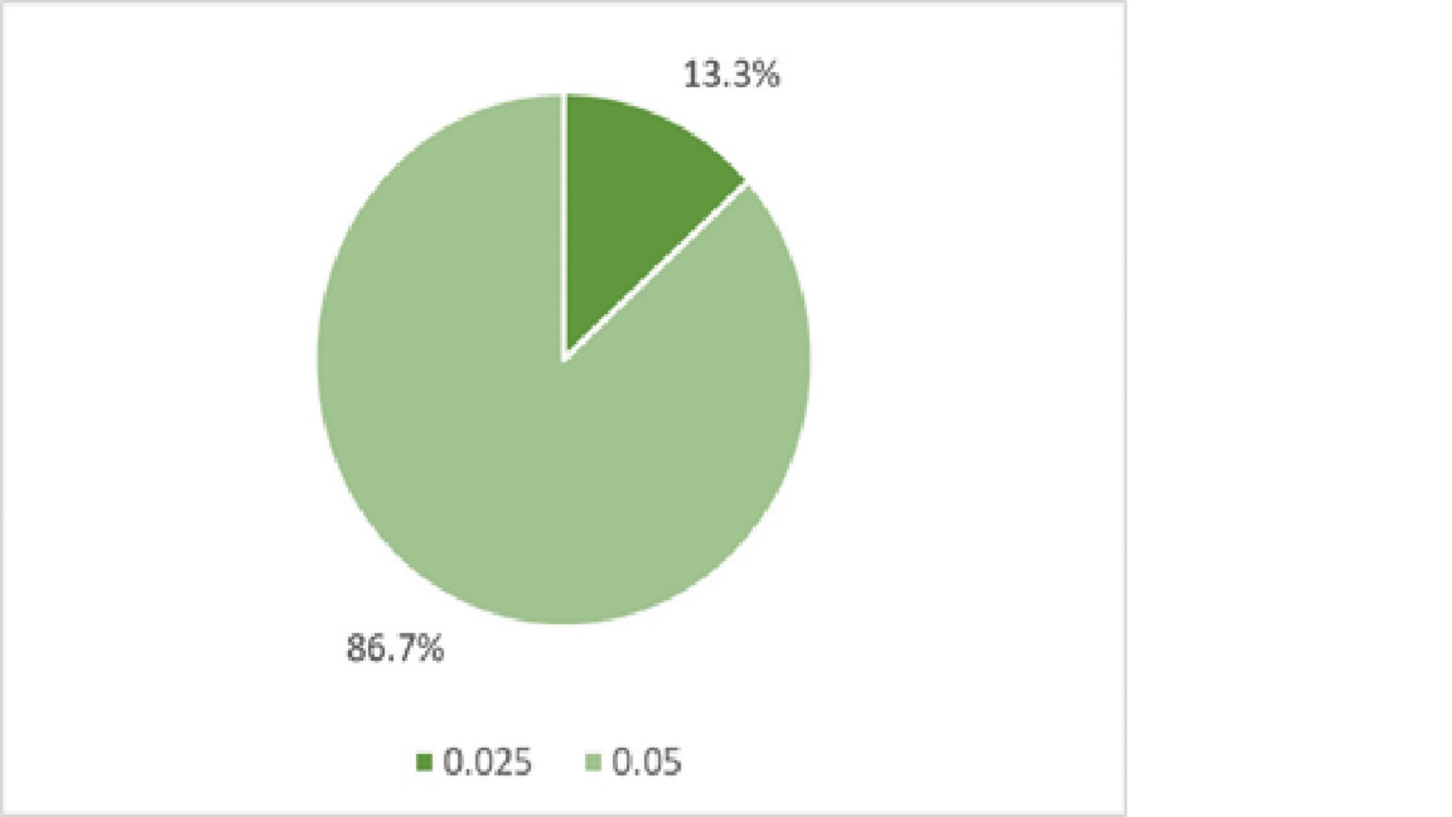
Distribution of type I error values across included RCTs evaluating surgical interventions for BPH. RCT: Randomized controlled trial; BPH: Benign prostatic hyperplasia.

A total of 56 randomized controlled trials were included in the final review. Of the 56 included studies, 30 (53.6%) reported conducting a sample size calculation, and 26 (46.4%) did not, as shown in Table 2. Similarly, power estimation was reported in 30 studies (53.6%). Type I errors were reported in 30 studies (53.6%). Every study that reported a sample size calculation also reported power estimation and the type I error threshold. A conventional type I error threshold of 0.05 was adopted in most cases (26 studies; 86.7%), whereas a smaller subset (4 studies; 13.3%) reported using a more stringent threshold of 0.025, as shown in Figure 2. A total of 25 studies documented the MCID; however, the reporting format varied considerably. While some studies reported the MCID as absolute numerical values, others expressed it as percentages, International Prostate Symptom Score (IPSS) thresholds, or maximum urinary flow rate (Qmax). This heterogeneity limited comparability across studies and precluded the possibility of conducting a pooled quantitative synthesis. Importantly, the variability in MCID definitions also complicates the interpretation of clinical significance; what one trial considers a meaningful symptom reduction may not be directly comparable to another, making it difficult to establish consistent benchmarks of treatment efficacy across BPH interventions.

**Table 2:** Characteristics of RCTs included in the systematic review, summarizing reporting practices for SSC, statistical power, type I error, and MCID. SSC: Sample size calculation; MCID: Minimal clinically important difference; RCT: Randomized controlled trial.

Variables	Yes (%)	No (%)
Sample size calculation reported	30 (53.6%)	26 (46.4%)
Power reported	30 (53.6%)	26 (46.4%)
Type I error reported	30 (53.6%)	26 (46.4%)
MCID reported	25 (44.6%)	31 (55.4%)

As shown in Table 3, in the earlier decades (1991-2000 and 2001-2010), reporting of sample size calculations was infrequent, observed in only 1 study (33.3%) and 2 studies (22.2%), respectively. However, from 2011 onward, the proportion of studies reporting SSC increased substantially, with 16 studies (64%) in 2011-2020 and 11 studies (57.9%) in 2021-2025. This trend suggests that recent decades have seen greater attention paid to methodological transparency.

**Table 3:** Trends in the reporting of SSC in RCTs of surgical interventions for BPH across different publication periods. SSC: Sample size calculation; RCT: Randomized controlled trial; BPH: Benign prostatic hyperplasia. Values are presented as frequency (percentage) of studies reporting SSC within each time period.

Years	Total studies	Yes (%)	No (%)
1991-2000	3	1 (33.3%)	2 (66.7%)
2001–2010	9	2 (22.2%)	7 (77.8%)
2011-2020	25	16 (64.0%)	9 (36.0%)
2021-2025	19	11 (57.9%)	8 (42.1%)

**Figure 3:**
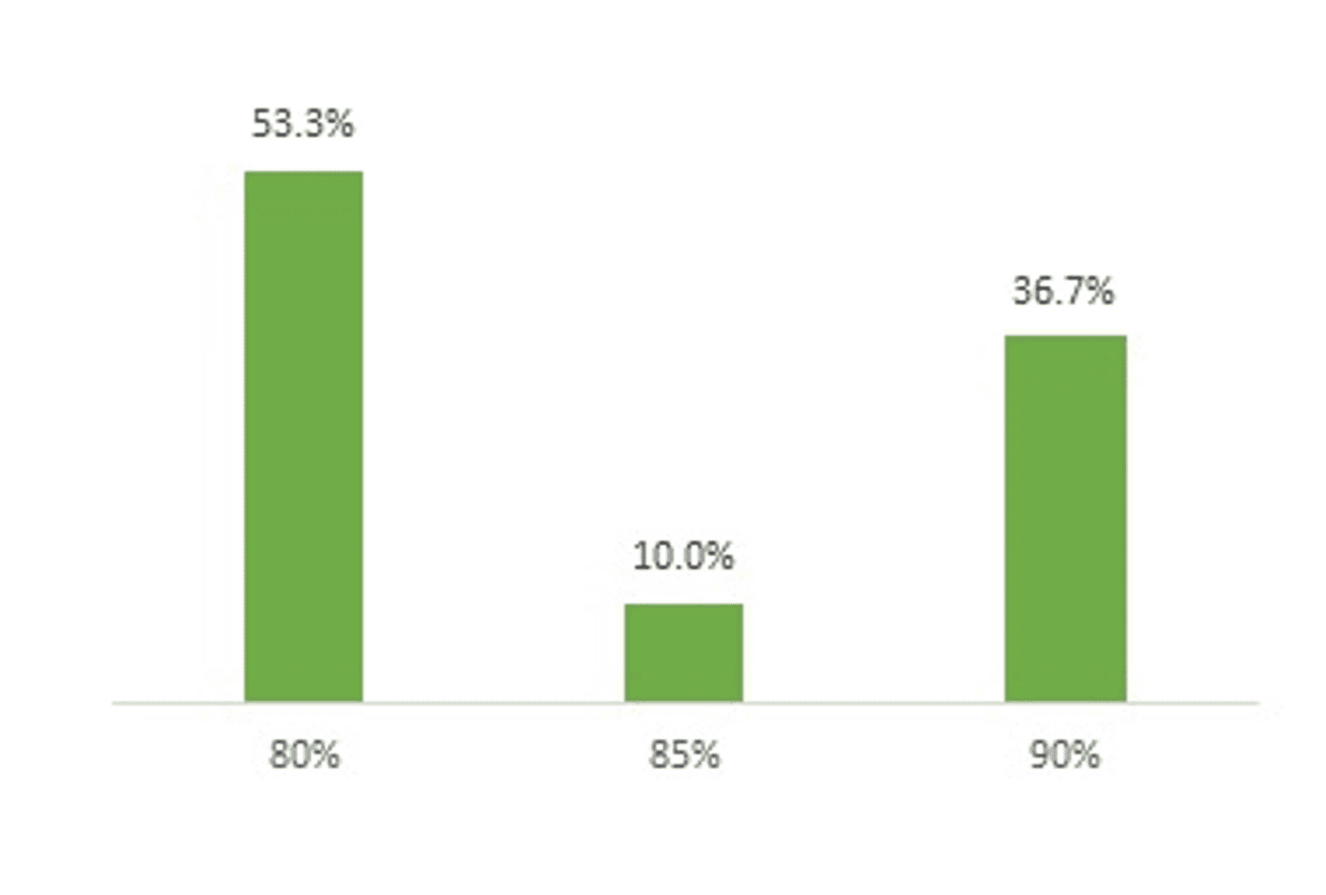
Distribution of reported statistical power values across included RCTs. RCT: Randomized controlled trials.

Among the trials that reported SSC, power values were specified in nearly all cases. As shown in Figure 3, slightly more than half of the studies (53.3%) adopted the conventional threshold of 80%, whereas 36.7% targeted a more stringent power of 90%, and 10% reported an intermediate value of 85%. This distribution suggests that while most researchers adhered to conventional practice, a notable proportion aimed for increased sensitivity in detecting clinically meaningful differences.

To investigate whether SSC reporting varied according to the type of intervention, the included studies were grouped based on the treatments under comparison. In accordance with the eligibility criteria, all trials contained at least one surgical intervention arm. For the purposes of analysis, standard surgery was defined as conventional operative procedures, including TURP and open prostatectomy. nMIST encompassed laser-based techniques such as HoLEP, ThuLEP, aquablation, and PVP. Sham interventions were categorized separately and referred to placebo-like surgical procedures performed for blinding without any expected therapeutic benefit. Finally, non-surgical management included pharmacological therapies or conservative strategies, such as watchful waiting and lifestyle modification. This classification allowed for a clear comparison of how SSCs were reported among the traditional, innovative, and control intervention groups.

When studies were stratified by intervention type, SSC reporting showed marked variation, as shown in Table 4. Trials comparing standard surgery with nMIST reported SSC in 69.6% of cases, whereas trials comparing nMIST with nMIST reported SSC in 56.3% of cases. Trials comparing standard surgery with standard surgery had lower rates (33.3%), whereas none of the studies comparing nMIST with sham or standard surgery with non-surgical management reported SSC. Overall, SSC reporting was most consistent in studies that included at least one novel minimally invasive therapy arm. When the categories were collapsed into nMIST versus non-nMIST, as shown in Table 5 using a two-by-two contingency table, 25 (62.5%) of nMIST trials reported SSC compared with 5 (31.3%) of non-nMIST trials. This difference was statistically significant (χ²(1) = 4.49, p = 0.034), with the odds of SSC reporting being higher in the nMIST trials (OR 3.67, 95% CI 1.07-12.62). These findings suggest that newer technologies are subject to greater methodological rigor, potentially reflecting stricter regulatory oversight, industry sponsorship, and increased peer-review scrutiny. However, given the small number of studies in some categories (e.g., nMIST/sham), these results should be interpreted cautiously, as individual data points may not reliably reflect broader reporting practices.

**Table 4:** Reporting of SSC in RCTs of surgical interventions for BPH stratified by intervention type. SSC: Sample size calculation; RCT: Randomized controlled trial; BPH: Benign prostatic hypertrophy; nMIST: Novel minimally invasive surgical therapy.

Intervention Categories	Total Studies	Yes (%)	No (%)
nMIST / nMIST	16	9 (56.3%)	7 (43.7%)
nMIST / Sham	1	0 (0%)	1 (100%)
Standard surgery / Non-surgical	1	0 (0%)	1 (100%)
Standard surgery / nMIST	23	16 (69.6%)	7 (30.4%)
Standard surgery / Standard surgery	15	5 (33.3%)	10 (66.7%)

**Table 5:** Two-by-two contingency table summarising the reporting of SSC in RCTs of surgical interventions for BPH, comparing nMIST-involved trials versus non-nMIST trials. SSC: Sample size calculation; RCT: Randomized controlled trial; BPH: Benign prostatic hyperplasia; nMIST: Novel minimally invasive surgical therapy.

Intervention Categories	SSC Yes	SSC No	Total
nMIST	25 (62.5%)	15 (37.5%)	40
Non-nMIST	5 (31.3%)	11 (68.7%)	16
Total	30	26	56

During the utilization of the Cochrane Risk of Bias 2 tool, each domain was rated as low risk, some concerns, or high risk. Most studies indicated a low risk in relation to the selection of reported results, but some concerns were noted in the area of outcome measurement. Overall, 12 (21.4%) of the studies were assessed as having a low risk of bias; 40 (71.4%) were assessed as showing some concerns/equivocal; and 4 (7.1%) were at high risk of bias, as shown in Table 6.

**Table 6:** Risk of bias assessment for included RCTs. RCT: Randomized controlled trial; ROB: Risk of bias.

Domain	Low	Some Concerns	High
Randomization	38	18	-
Deviation	48	7	1
Missing Data	38	15	3
Measurement	19	35	2
Reporting	51	5	-
Overall ROB	12 (21.4%)	40 (71.4%)	4 (7.1%)

Discussion

This systematic review examined the reporting practices related to sample size calculation and power estimation in randomized controlled trials of surgical interventions for BPH. Although the methodological rigor of sample size planning is widely recognized as essential for ensuring adequately powered trials [10,13], our findings demonstrate that just over half of the included studies explicitly reported conducting a sample size calculation and specifying the power. This is consistent with previous systematic evaluations of clinical trial reporting, which have noted persistent deficiencies in the transparent reporting of these parameters [18].

The reporting of alpha values demonstrated a high degree of uniformity, with the vast majority of studies adhering to the conventional 0.05 threshold value. Only a small minority adopted a more conservative value (0.025). This suggests that while power considerations may vary across trials, the selection of the type I error threshold remains largely standardized in the BPH literature. In contrast, power values were more heterogeneous, with approximately 50% of studies reporting the conventional 80% threshold, while a significant proportion opted for 90%. This trend may reflect the growing recognition of the need to reduce type II error risk in surgical research, although the use of intermediate values (e.g., 85%) highlights the variability in statistical planning practices. Overall, all articles that reported power used an acceptable value, with the majority adopting the conventional threshold of 80%, which is generally considered adequate for ensuring reliable results.

The reporting of the MCID is less consistent and often poorly standardized. While some studies reported the MCID in absolute terms, others expressed it as a percentage or used different clinical outcome measures, such as IPSS or Qmax. This lack of uniformity not only limits comparability across trials but also raises questions about how investigators conceptualize clinically meaningful changes. A possible way forward is the development of consensus-based reporting standards for MCID in BPH trials, similar to initiatives in oncology and cardiology, where standardized definitions of clinically important differences have been proposed [78]. For instance, trialists could be encouraged to adopt disease-specific thresholds derived from validated patient-reported outcome measures, such as a three-point reduction in IPSS or a 2-3 mL/s increase in Qmax, which have previously been suggested as minimally important improvements in urology research [79]. In addition, aligning MCID reporting with established frameworks, such as the Core Outcome Measures in Effectiveness Trials (COMET) initiative, could help harmonize the definitions of clinical significance across trials [80]. Standardization of the MCID would enhance interpretability, allow more robust meta-analyses, and strengthen the translation of trial findings into clinical practice.

The implications of this heterogeneity are extensive. Inconsistent definitions of MCID reduce the interpretability of trial results and make it difficult to synthesize evidence across studies. For example, a treatment effect deemed clinically meaningful in one trial may not meet the threshold in another trial, even when evaluating similar interventions and outcomes. This variability introduces challenges for meta-analyses, potentially leading to misleading conclusions or underestimation of the true clinical benefit. Therefore, establishing consensus thresholds for MCID in BPH, inform informed by validated patient-reported outcomes and anchored in clinically relevant changes such as IPSS reduction or Qmax improvement, is critical to improving the comparability and clinical applicability of future trials.

The improvement in SSC reporting over the past two decades reflects global calls for greater methodological transparency in clinical research [18]. International guidelines, such as the CONSORT statement, have emphasized the importance of sample size justification in randomized controlled trials, which may partly explain this upward trend. Nevertheless, SSC reporting has not yet reached universal adoption, indicating persistent gaps in methodological rigor. Similar trends have been noted in other surgical and non-surgical trials, where methodological rigor has improved but has not yet reached optimal standards [11]. This inconsistency highlights the continued need for stricter enforcement of reporting guidelines by journals and peer reviewers to enhance the credibility and reproducibility of RCTs on BPH surgery.

The analysis by intervention category indicated that SSC reporting was inconsistent across study types. Trials comparing nMIST with traditional approaches were more likely to report SSC (OR 3.67; 95% CI 1.07-12.62; p = 0.034), potentially reflecting heightened methodological scrutiny when evaluating new technologies. In contrast, trials evaluating established procedures, such as TURP or open prostatectomy, may have relied on historical assumptions or prior conventions, resulting in less frequent reporting. Another factor may be the influence of funding: industry-sponsored studies of new devices often face stricter regulatory oversight, which may compel more rigorous methodological disclosure, whereas investigator-initiated trials of standard procedures may not encounter the same demands [81]. However, interpretation should be cautious, as the analysis was exploratory and post hoc, the confidence interval was wide, and it barely exceeds the null value, suggesting that the observed difference may reflect an exploratory trend rather than a definitive association. This imprecision likely arises from the relatively small number of trials within each subgroup and underscores the need for caution in interpreting the apparent association between intervention type and methodological reporting.

These findings suggest that the quality of SSC reporting may be influenced not only by publication year but also by the type of intervention and context of trial sponsorship. To address these discrepancies, future research should encourage uniform adherence to methodological reporting standards regardless of the intervention type. Journals and peer reviewers could play a pivotal role by mandating full disclosure of SSC parameters across all BPH trials, while professional urological societies might issue field-specific recommendations emphasizing transparent and consistent reporting in both conventional and novel surgical trials.

## Conclusions

This systematic review demonstrates that although the reporting of SSC and power estimations in RCTs of surgical treatments for BPH has improved over the past four decades, considerable gaps remain. More recent trials have shown higher compliance with methodological standards; however, inconsistencies persist in the reporting of key parameters, particularly the MCID. Moreover, reporting practices varied by intervention type, with trials involving nMIST more frequently providing methodological details than those assessing established procedures. This exploratory observation may indicate variability in methodological reporting practices across intervention types.

These findings underscore the importance of strengthening transparency and consistency in methodological reporting in all BPH trials. Rigorous and standardized approaches to sample size planning and outcome reporting will enhance the reliability, comparability, and clinical utility of future evidence, thereby supporting more robust decision-making in the management of BPH.
